# Single Cell Transcriptome and Surface Epitope Analysis of Ankylosing Spondylitis Facilitates Disease Classification by Machine Learning

**DOI:** 10.3389/fimmu.2022.838636

**Published:** 2022-05-13

**Authors:** Samuel Alber, Sugandh Kumar, Jared Liu, Zhi-Ming Huang, Diana Paez, Julie Hong, Hsin-Wen Chang, Tina Bhutani, Lianne S. Gensler, Wilson Liao

**Affiliations:** ^1^ Department of Electrical Engineering and Computer Sciences, University of California at Berkeley, Berkeley, CA, United States; ^2^ Department of Dermatology, University of California at San Francisco, San Francisco, CA, United States; ^3^ Division of Rheumatology, Department of Medicine, University of California at San Francisco, San Francisco, CA, United States

**Keywords:** ankylosing spondylitis, spondyloarthritis, single cell sequencing, CITE-seq, genomics, machine learning

## Abstract

Ankylosing spondylitis (AS) is an immune-mediated inflammatory disorder that primarily affects the axial skeleton, especially the sacroiliac joints and spine. This results in chronic back pain and, in extreme cases, ankylosis of the spine. Despite its debilitating effects, the pathogenesis of AS remains to be further elucidated. This study used single cell CITE-seq technology to analyze peripheral blood mononuclear cells (PBMCs) in AS and in healthy controls. We identified a number of molecular features associated with AS. CD52 was found to be overexpressed in both RNA and surface protein expression across several cell types in patients with AS. CD16^+^ monocytes overexpressed *TNFSF10* and IL-18Rα in AS, while CD8^+^ T_EM_ cells and natural killer cells overexpressed genes linked with cytotoxicity, including *GZMH, GZMB*, and *NKG7*. Tregs underexpressed CD39 in AS, suggesting reduced functionality. We identified an overrepresented NK cell subset in AS that overexpressed CD16, CD161, and CD38, as well as cytotoxic genes and pathways. Finally, we developed machine learning models derived from CITE-seq data for the classification of AS and achieved an Area Under the Receiver Operating Characteristic (AUROC) curve of > 0.95. In summary, CITE-seq identification of AS-associated genes and surface proteins in specific cell subsets informs our understanding of pathogenesis and potential new therapeutic targets, while providing new approaches for diagnosis *via* machine learning.

## Introduction

Affecting approximately 0.52-0.55% of the US population, ankylosing spondylitis (AS) is a chronic inflammatory disease that targets sacroiliac joints, spine, peripheral joints and entheseal attachment sites (1). In more severe cases, AS can cause fibrosis and calcification, resulting in ankylosis of the sacroiliac joints and spine (2). AS is part of a broader group of rheumatologic diseases commonly characterized by inflammatory back pain, enthesitis, and dactylitis known as spondyloarthritis (3). Extra-musculoskeletal manifestations of AS include acute anterior uveitis and psoriasis, and comorbidities include cardiovascular disease and osteoporosis (4–6). Additionally, it has been demonstrated that non-rheumatologists do not consider the diagnosis of AS in patients presenting with back pain, creating a delay in diagnosis and treatment (7). The most common method of ankylosing spondylitis diagnosis and classification is the modified New York Classification Criteria, which involves both radiological criterion, such as biliteral sacroiliitis grade ≥ II, and clinical criteria, such as limitation of chest expansion relative to values normal for age and sex (8).

Previous studies have pointed to the significance of genetic and immunological factors in AS. In particular, the major histocompatibility complex class I allele HLA-B*27 was shown to be present in the majority of patients with AS, serving as a key biomarker for AS and determining a patient’s susceptibility to the disease (9). Nevertheless, although the heritability of the susceptibility of AS is estimated to be around 90%, the contribution of HLA-B*27 to this heritability is only roughly 20%, pointing to the presence of other genetic factors (10, 11). Other known genes contributing to AS include *ERAP1* and *ERAP2*. The IL-23/IL-17 axis has been shown to play a vital role in driving the inflammation behind AS.

Several cell types in the peripheral blood of patients with AS are thought to be involved in the pathogenesis of AS. Natural killer (NK) cells, while not expanded in AS (12), have been shown to respond to HLA-B27 *via* the KIR3DL1 receptor (13). CD4^+^ T cells increase production of IP-10/CXCL10, which recruits Th1 cells that then amplify the inflammatory response *via* secretion of IFN-γ and TNF-α (14). Th17 cells have also been observed to play a key role in the pathogenesis of AS through the production of several inflammatory cytokines, such as IL-17 (15).

In this study, we used single-cell technology to help identify cellular composition differences as well as differentially expressed genes, proteins, and pathways in the peripheral blood mononuclear cells (PBMCs) of patients with AS. We utilized a multi-omic approach, surveying both transcriptome and cell surface proteins involved in this disease, and evaluated the diagnostic potential of these biomarkers using machine learning models to identify AS patients. To our knowledge, this is the first attempt to use machine learning and single cell transcriptome data to classify AS.

## Methods

### Patient Recruitment and Sampling

Patients with ankylosing spondylitis (n=10; 6 male, 4 female) were enrolled from the rheumatology clinics at the University of California San Francisco (UCSF), with a board-certified rheumatologist confirming the clinical diagnosis of AS using the modified New York classification criteria. Nine of the ten AS subjects were not on any biologic therapy, while one AS subject was on ustekinumab for his concomitant Crohn’s disease. Healthy controls (n=29), who did not have any inflammatory skin disease or autoimmune disease, were enrolled from the San Francisco Bay Area. All subjects gave written, informed consent under IRB approval 10-02830 from the University of California San Francisco. Detailed patient information is provided in **Supplementary Table 1**. Peripheral blood was collected from each subject in Vacutainer ACD tubes. PBMCs were isolated using a standard Ficoll method and stored in liquid nitrogen.

### Sample and Library Preparation

#### Single Cell Libraries

500 µL thawed PBMCs from each subject were added to 10 mL EasySep (StemCell Technologies, Cat. 20144) and centrifuged (300G, 5 min, room temperature). Extracellular nucleic acids were digested by resuspending cell pellets in 1 mL of buffer made from 18 mL EasySep and 21 µL Benzonase Nuclease (MilliporeSigma, Cat. 70664) and incubating (15 min, room temperature). Nuclease-treated cell-suspensions were then filtered through a 40 µm Flowmi Cell Strainer (Bel-Art, Cat. H13680-0040), centrifuged (300G, 5 min, room temperature), and finally resuspended in 100 µL EasySep buffer. Cell counting was performed on 1:100 dilutions of final cell suspensions stained with 0.4% trypan blue using a Countess I FL Automated Cell Counter (Thermo Fisher Scientific).

#### Cell Surface Staining

Antibody staining of cell surface proteins was performed according to the Totalseq-A protocol (https://www.biolegend.com/en-us/protocols/totalseq-a-antibodies-and-cell-hashing-with-10x-single-cell-3-reagent-kit-v3-3-1-protocol) with modifications as follows. A pooled suspension containing 100,000 cells from at most 20 subjects at a time was centrifuged (300G, 5 min, 4°C) and resuspended in 100 µL Cell Staining Buffer (BioLegend, Cat. 420201) and incubated (10 min, 4°C) with 10 µL Human TruStain FcX™ Fc Blocking Solution (BioLegend, Cat. 422301). Cells suspensions were then stained (30 min, 4°C) with 100 µL TotalSeq antibody cocktail (**Supplementary Table 2**) and divided into two 105 µL aliquots. Each aliquot was washed 3 times by resuspending in 15 mL Cell Staining Buffer and centrifuging (300G, 5 min, 4°C). Washed cells were then resuspended in 150 µL 10% FBS in PBS, recombined, and filtered again with a 40 µm Flowmi Cell Strainer. Cell viability was measured with 10 µL of filtered cells by adding 10 µL 0.4% Trypan Blue and manual counting with a hemocytometer. Cell density was adjusted to 2,500 cells/µL and run on the Chromium Controller (10X Genomics) using the Single Cell 3’ v3.1 Assay (10X Genomics) with a target of 50,000 cells per reaction.

#### Library Preparation

Gene expression cDNA libraries were prepared using according to the manufacturer’s instructions (https://assets.ctfassets.net/an68im79xiti/1eX2FPdpeCgnCJtw4fj9Hx/7cb84edaa9eca04b607f9193162994de/CG000204_ChromiumNextGEMSingleCell3_v3.1_Rev_D.pdf), with 12 cycles of PCR amplification. Libraries for antibody-derived tags (ADT) from feature barcoding antibodies were prepared by repeating size purification on the supernatant obtained from the prior size purification of gene expression cDNA libraries (Step 2.3.d in the manufacturer’s instructions above), using 7:8 volumetric ratio of 2.0X SPRIselect reagent (Beckman Coulter, Cat# B23317) to sample. Indexing amplification was performed using Kapa Hifi HotStart ReadyMix (Kapa Biosystems, Cat# KK2601) and TruSeq Small RNA RPI primers (Illumina) with the following thermocycling conditions: (I) 98°C, 2 min; (II) 15 × (98°C, 20 sec; 60°C, 30 sec; 72°C, 20 sec); (III) 72°C, 5 min. Size purification was then repeated on amplified libraries using a 5:6 volumetric ratio of 1.2X SPRIselect reagent to sample. Libraries were quantified using a Bioanalyzer 2100 (Agilent) and sequenced on a Novaseq 6000 (Illumina).

#### Genotyping

DNA for genotyping was extracted from whole blood using the DNeasy blood and tissue kit (Qiagen, Cat. 69504). Extracted DNA was genotyped on the Affymetrix UK Biobank Axiom Array (ThermoFisher) using a GeneTitan Multi-Channel Instrument (Applied Biosystems).

### Genotype Data Processing

SNPs were called using Analysis Power Tools 2.10.2.2 (Affymetrix, https://www.affymetrix.com/support/developer/) The resulting genotype vcfs were scanned with snpflip (https://github.com/biocore-ntnu/snpflip) using the GRCh37 build of the human genome reference sequence maintained by the University of California, Santa Cruz (http://hgdownload.cse.ucsc.edu/goldenPath/hg19/bigZips/hg19.fa.gz) to identify reversed and ambiguous-stranded SNPs, which were flipped and removed (respectively) using Plink 1.90 (http://pngu.mgh.harvard.edu/purcell/plink/) (16), and the remaining sites were sorted using Plink 2.00a3LM (www.cog-genomics.org/plink/2.0/) (17). This SNP data was then augmented with additional sites imputed by the Michigan Imputation Server (https://imputationserver.sph.umich.edu) (1000G Phase 3 v5 GRCh37 reference panel, rsqFilter off, Eagle v2.4 phasing, EUR population). SNP positions were translated to GRCh38 coordinates using the ‘LiftoverVcf’ command of Picard 2.23.3 (http://broadinstitute.github.io/picard/). Finally, Vcftools 0.1.13 (18) was used to exclude non-exonic SNPs and SNPs with minor allele frequency *<* 0.05.

### Single Cell Data Processing

Raw RNA and ADT fastqs for each Chromium library were respectively aligned to the GRCh38 human genome reference and the antibody-tag reference (**Supplementary Table 2**) using Cell Ranger 3.1.0 (10X Genomics) using default settings to obtain RNA and matched ADT (if available) count matrices for all barcodes representing non-empty droplets.

#### Cell Demultiplexing, Doublet Removal, and Annotation

Within each RNA count matrix, the subject of origin for all droplet barcodes was determined by using ‘demuxlet’ (19), as implemented in the ‘popscle’ suite (https://github.com/statgen/popscle) to imputation-augmented exonic SNP genotypes described above, and doublets detected between different individuals were excluded. The count matrices for each Chromium library were then loaded into R for analysis using the ‘Seurat’ 4.0.3 (20) R package, and the ‘DoubletDecon’ 1.1.6 R package (21) was used to further remove doublets formed by different cells within the same individual.

Annotations for each droplet barcode were determined by submitting raw RNA count matrices to Azimuth (https://azimuth.hubmapconsortium.org/) (20) for annotation with “celltype.l2” labels from the Human PBMC reference from Hao et al. (20).

#### Cell and Feature QC

We performed filtering of cells based on both RNA and ADT data by retaining cells with total RNA unique molecular identifiers (UMIs) between 500 and 10,000, total RNA features ≥ 200, percent mitochondrial and ribosomal protein reads in RNA ≤ 15% and 60% (respectively), total ADT features ≤ 260, and percent ADT reads mapping to 9 isotype control antibodies < 2%. In the RNA matrices of the resulting data, we further removed features (genes) with no detectable UMIs across the cells of all matrices. These matrices were finally merged together into a combined matrix of RNA data for all cells. In the ADT matrices, we further removed features corresponding to the 9 isotype controls and 15 features observed to have expression inconsistent with annotated cell types (**Supplementary Table 2**).

#### Intra-Cell Type Differential Feature Analysis and Clustering

To identify differentially expressed genes (DEGs) and proteins (DEPs), the Seurat object containing ADT and RNA expression from the QC’d dataset (see section ‘Cell and feature QC’ above) was subsetted by Azimuth-annotated cell type using ‘SplitObject’. For each resulting Seurat object containing cells of a particular type, we performed normalization on RNA and ADT expression using SCTransform and CLR, again adjusting for total counts and total features in each cell (using the ‘vars.to.regress’ parameter). Differential gene expression between disease statuses as well as between clusters (see section ‘Intra-cell type clustering’) was then calculated on SCTransform-normalized counts using the negative binomial test (test.use = “negbinom” in Seurat). Genes with both Bonferroni-corrected p-value < 0.05 and absolute log fold change > 0.20 were considered significant. Differential protein analysis was performed similarly, except with the Wilcoxon test (test.use = “wilcox” in Seurat) on CLR-normalized, mean-centered and scaled ADT data (within the ‘scale.data’ slot of the Seurat object) only for cells with measured ADT data. The expression of DEGs and DEPs was compared between batches; DEGs and DEPs that had a clear overexpression in a small subset of the batches, such as *MTRNR2L12*, were filtered out. Pathway analysis was performed on differentially expressed genes *via* the ‘gprofiler2’ R package (22) against the Gene Ontology (GO), KEGG, and Reactome databases. For identification of transcription factors, gprofiler2 was also run against the TRANSFAC database. Afterwards, the p values returned by gprofiler2 were adjusted to FDR values for each database.

To identify phenotypic clusters within cell types, the RNA expression data for a cell type was first corrected for batch effects by first subsetting the raw count matrix by the cells within each sequencing batch. SCTransform was run individually for each count matrix, and the resulting SCT expression matrices were reintegrated into a single matrix (see section ‘Data integration’). PCA was performed on the integrated SCT matrix, and the first 30 PCs were used to construct a shared nearest-neighbor network using the ‘FindNeighbors’ function. The network was then used to identify clusters with the ‘FindClusters’ function, using a resolution of 0.6. UMAPs were also generated from the first 30 PCs using the ‘RunUMAP’ function.

#### Data Integration

Integration of SCT expression data from two or more single-cell datasets was performed according to the Seurat data integration protocol (https://satijalab.org/seurat/articles/integration_introduction.html#performing-integration-on-datasets-normalized-with-sctransform-1). Briefly, ‘SelectIntegrationFeatures’ was used to select a common set of 3,000 genes most consistently variable among the individual SCT matrices, and ‘PrepSCTIntegration’ was then used to prepare reduced SCT expression matrices for just these genes. PCA was calculated for each reduced SCT matrix using ‘RunPCA’, and the first 50 principal components of this transformation were used to identify transcriptomically similar cells between each pair of reduced SCT matrices using ‘FindIntegrationAnchors’, with ‘reduction’ set to ‘rpca’. Finally, an integrated SCT matrix was calculated using ‘IntegrateData’.

### Machine Learning Model Development

The input dataset for machine learning classification of AS and healthy subjects consisted of, for each subject, the means of sctransform-normalized, centered, and scaled expression of each feature in the set of cell-type-specific differentially expressed genes and proteins, calculated across that subject’s cells within the corresponding cell types. These mean expression data for 39 subjects (29 healthy and 10 AS) were randomly assigned by a 50:50 ratio into training (healthy = 15 and AS = 5) and test (healthy = 14 and AS = 5) sets for ML model building and evaluation.

We first performed ensemble-based feature selection using the EFS-MI method (23) where subsets of the starting feature set predicted to be informative by four different ML algorithms (Feed Forward and Backward selection, Recursive RF, SVMRadial, and NNET) were combined and sorted by prediction potential classification rank. We selected the top twenty features to train nine ML algorithms based on linear, non-linear, and ensemble models provided by the ‘caret’ R package. Five-fold cross validation, repeated twice, was performed on the training set using each ML algorithm, and resulting models were evaluated on the test set. All essential tuning parameter were optimized with bootstrap = TRUE. For random forest (RF) models, the maximum number of tree splits in each step fixed a max_depth = (50, 80, 100, 150, 300), the maximum feature selected as auto (max_features = ‘auto’), and error was minimized through impurity value (min_impurity_decrease = c(0.0, 0.02, 0.1, 0.5). Further, a minimum tree split per leaf in each step (min_samples_leaf = (1 to 10) while maximum generation of trees (n_estimator = 20) was considered, other parameters kept as a default for RF. For SVMRadial, we tuned cost and sigma factor for correct classification. In avNNet and Naïve Bayes, we used TRUE kernel, decays, and their size as a tuning parameter. Model performance and robustness were evaluated based on classification statistics that include accuracy, area under receiver operating characteristic curve (AUROC), specificity, sensitivity, F1 score (harmonic mean of precision and recall), and balanced accuracy (kappa).

To check for model bias due to potentially shared information between test and training subsets (which are derived from data normalized by ‘sctransform’ over cells from all subjects), we regenerated the input dataset using an alternative normalization approach that aggregates single-cell data only within the cells of each subject into a representative expression profile for each subject. Specifically, the expression value for a gene [or protein] feature for a given subject was calculated as


$$\text{ln}\left(\frac{\text{feature counts across all cells in subject}}{\text{total counts across all cells in subject}}\;\times \;\text{scaling factor}\right)$$

where the scaling factor was chosen to be near the maximum number of counts across all subjects (10^7^ for RNA, 5 × 10^5^ for ADT). Training and testing of models was performed as above to evaluate accuracy and kappa. AUROC was also calculated for select models trained using 10-fold cross validation with 10 repeats.

## Results

### Identifying Significant Peripheral Blood Mononuclear Cell Types in AS

**Figure 1:**
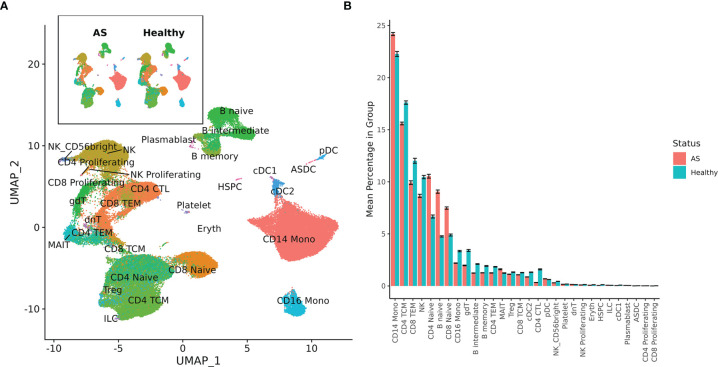
Cell types among AS and healthy PBMCs. **(A)** UMAP of 30 cell types in AS and healthy PBMCs based on RNA expression. **(B)** Mean percentage of each cell type within the total PBMCs from each subject, averaged across AS and healthy cohorts. Error bars represent standard error of the mean. When tested for statistical significance using the Wilcoxon rank-sum test, CD4+ cytotoxic T cells and hematopoietic stem and progenitor cells were significantly underexpressed in AS.

Single-cell sequencing of PBMCs from 10 patients with AS and 29 healthy controls yielded transcriptome profiles of 98,884 cells (19,348 cells from patients with AS, 79,536 from the healthy controls). Single-cell RNA and ADT analysis was conducted on 59,585 of these cells and just single-cell RNA analysis was conducted on the other 39,299 cells (**Supplementary Figure 1**). Reference-based categorization of AS and healthy PBMCs into 30 unique cell types (**Figure 1A**) revealed a significantly lower abundance of CD4^+^ cytotoxic T cells and hematopoietic stem and progenitor cells (HSPCs) in AS subjects (**Figure 1B**).

### Differentially Expressed Genes and Pathways Associated With AS in Circulating Immune Populations

**Figure 2:**
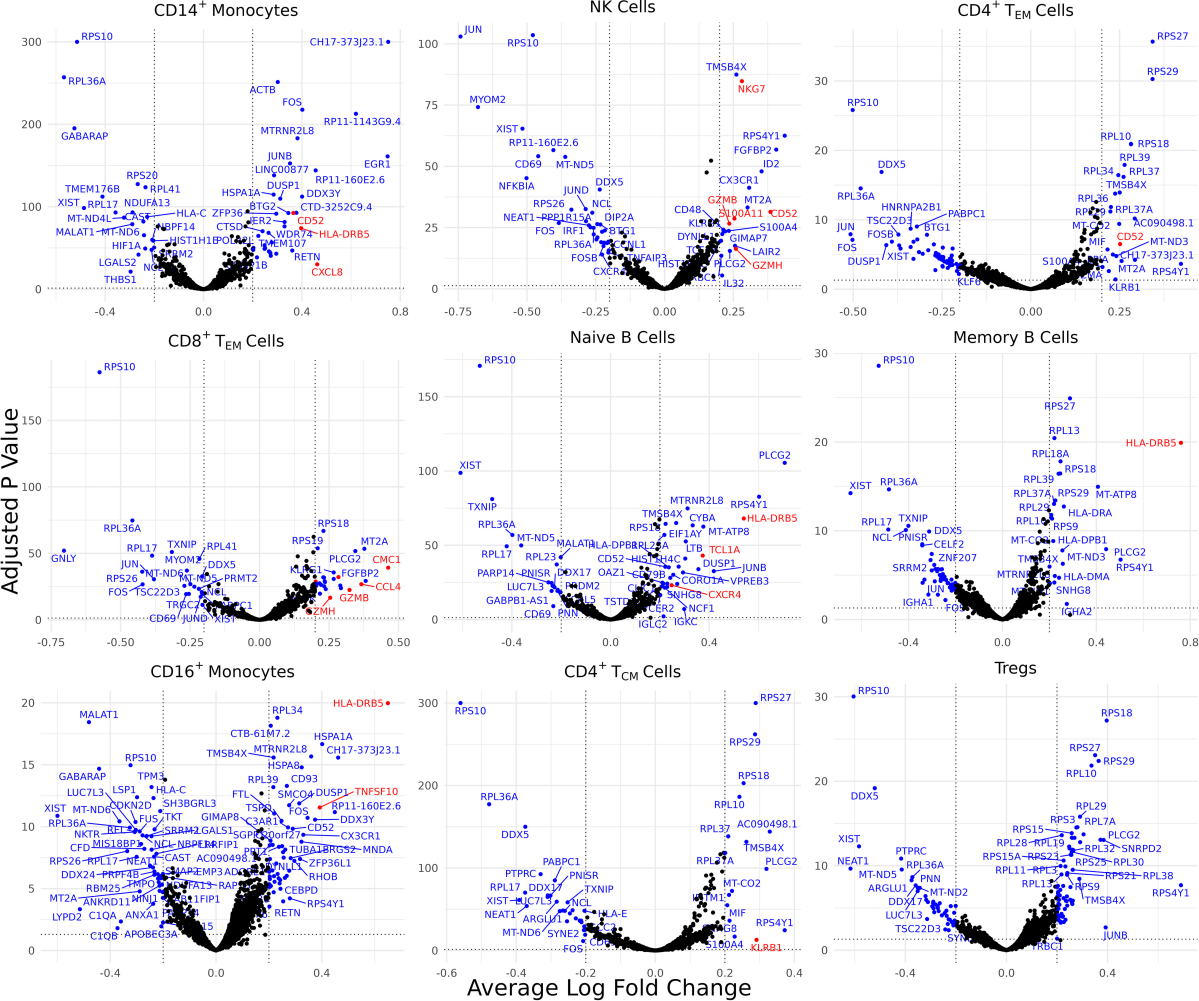
Cell type-specific DE genes between AS and healthy subjects. Volcano plots showing the DEGs for 9 cell types based on a Bonferroni-adjusted p-value < 0.05 and absolute average log 2 fold change ≥ 0.20. Blue points represent statistically significant genes and genes mentioned in the paper are highlighted in red.

Differentially expressed genes (DEGs) identified for each of the 30 identified cell types varied from 0 for several cell types to 88 for CD4^+^ naïve T cells, resulting in 898 total DEGs across all cell types (**Supplementary Figure 2A**). Of these, 9 cell types with a high number of biologically significant DEGs are shown in **Figure 2**, with the rest found in **Supplementary Table 3**.

Biologically relevant genes were identified across several cell types (**Figure 2**). *CD52* was overexpressed in AS CD14^+^ monocytes, natural killer cells, and CD4^+^ effector memory T (T_EM_) cells. CD8^+^ T_EM_ cells and natural killer cells overexpressed genes linked with cytotoxicity, including *GZMH, GZMB*, and *NKG7*. *HLA-DRB5* was overexpressed in the CD14^+^ monocytes, naïve B cells, memory B cells, and CD16^+^ monocytes of AS patients. CD8^+^ T_EM_ cells also overexpressed *CMC1, CCL4*, and *CCL4L2* while natural killer cells overexpressed *S100A11*. We observed an upregulation of *CXCL8* in AS CD14^+^ monocytes and an upregulation of *TNFSF10* in CD16^+^ monocytes. CD4^+^ T_CM_ cells in patients with AS overexpressed *KLRB1*. Naïve B cells overexpressed *TCL1A* and *CXCR4*.

Comparison of inflammatory cytokines involved in AS, including *TNF, IL1B, IL17A, IFNG, IL23A, IL7R*, and *IL17F*, revealed comparable expression between AS and healthy subjects for each annotated cell type except for mucosal-associated invariant T (MAIT) cells, in which we observed a statistically significant decrease in *IL7R* expression in AS cells (**Supplementary Table 3**) that was also reflected in cell surface expression of IL-7R*α* protein in the ADT data (**Supplementary Table 4**).

### Proteomic Analysis Reveals Inflammatory Cell Surface Proteins in AS

**Figure 3:**
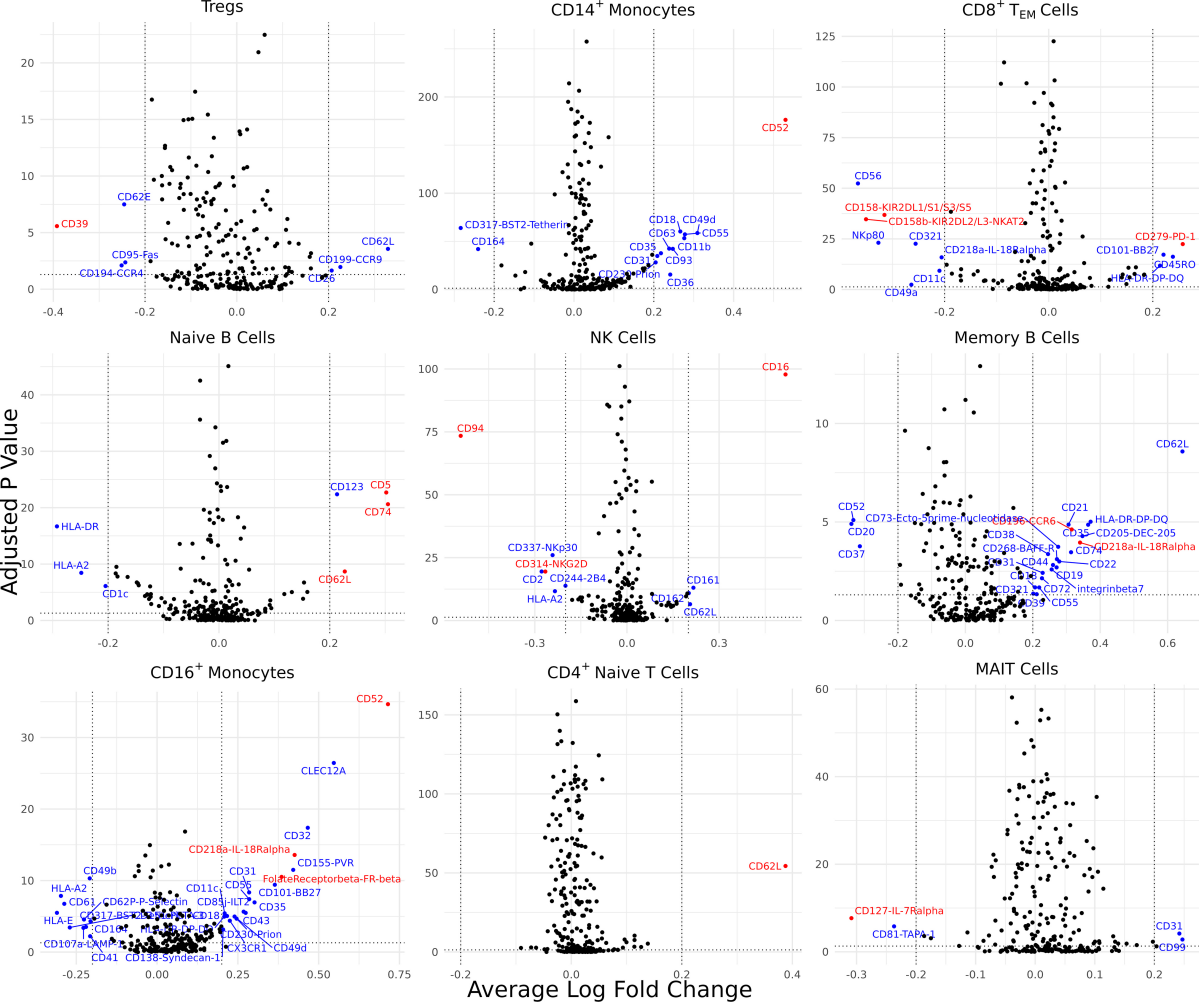
Cell type-specific DE proteins between AS and healthy subjects. Volcano plots showing the DEPs for 9 cell types based on cells with measured ADT data; blue points represent statistically significant proteins and proteins mentioned in the paper are highlighted in red. DEPs were identified based on a Bonferroni-adjusted p-value < 0.05 and absolute average difference ≥ 0.20.

Differentially expressed cell surface proteins (DEPs) were calculated for each cell type (0 – 24 features, **Supplementary Figure 2B**) between AS and healthy cells with ADT data measuring 258 cell surface proteins (**Figure 3**). Tregs in AS underexpressed CD39. CD14^+^ monocytes and CD16^+^ monocytes in AS overexpressed CD52. CD8^+^ T_EM_ cells overexpressed PD-1, KIR2DL2/L3 and KIR2DL1/S1/S3/S5. Natural killer cells in AS overexpressed CD16 but underexpressed CD94 and NKG2D. Memory B cells and CD16^+^ monocytes overexpressed IL-18R*α*. Memory B cells in AS also overexpressed CCR6 while naïve B cells overexpressed CD5 and CD74. Both CD4^+^ T_EM_ and MAIT cells in AS underexpressed IL7R*α*. CD16^+^ monocytes in AS overexpressed folate receptor *β* (FR-*β*).

### *De Novo* Clustering Reveals an NK Subset Associated With AS

Due to the overexpression of CD16 in AS NK cells, the proportion of CD16^+^ CD56^dim^ NK cells was compared between patients with AS and control patients *via* the Wilcoxon test, where it was found that CD16^+^ CD56^dim^ NK cells were significantly overrepresented in patients with AS (p = 0.006; **Supplementary Figure 3A**). To investigate whether there was a subset in NK cells driving this overexpression of CD16, we performed *de novo* clustering on NK cells (**Supplementary Figure 3B**). A cluster was identified in NK cells that was statistically overrepresented in patients with AS (**Supplementary Figure 3C**). This subset overexpressed CD16, CD38, and CD161 on the ADT level and *SPON2, NKG7, FGFBP2, KLRB1*, and *MYOM2* on the RNA level (**Supplementary Table 5** and **Supplementary Figure 3D**). *De novo* clustering was also performed on CD8^+^ T_EM_ cells, naïve CD8^+^ T cells, and CD14^+^ monocytes, however no subsets in any of these cell types were statistically overrepresented in AS.

### Gene Set Enrichment Analysis

To capture the relationships and shared pathways between differentially expressed genes in AS, we conducted functional enrichment analysis using gprofiler2 (**Supplementary Table 6**). Several pathways were significant at a nominal level (p < 0.05) but did not remain significant after FDR correction. CD14^+^ monocytes were observed to upregulate pathways related to IL-4 and IL-13 signaling, which were primarily constituted by *CXCL8, FOS*, and *JUNB*. Memory B cells upregulated pathways for Th17 cell differentiation, Th1/Th2 cell differentiation, and interferon-gamma-mediated signaling pathways.

To identify important transcription factors in our dataset, we conducted a separate gene set enrichment analysis using gprofiler2 and the TRANSFAC database. We found a set of transcription factors that were significant at the nominal level but did not remain significant after FDR correction. To help provide evidence for our results, we compared our identified transcription factors with a past study that used ATAC-seq on AS PBMCs (24). There, both our dataset and the ATAC-seq dataset found a statistically significant enrichment of GCM1, ETS1, ETV4, and ELF1 in CD4^+^ T cells. The NK cell subset that was found to be overrepresented in AS during *de novo* clustering upregulated NK cell mediated cytotoxicity (p = 0.003); however, this pathway was no longer statistically significant after correction (FDR = 0.2).

### Machine Learning Classification of AS

**Figure 4:**
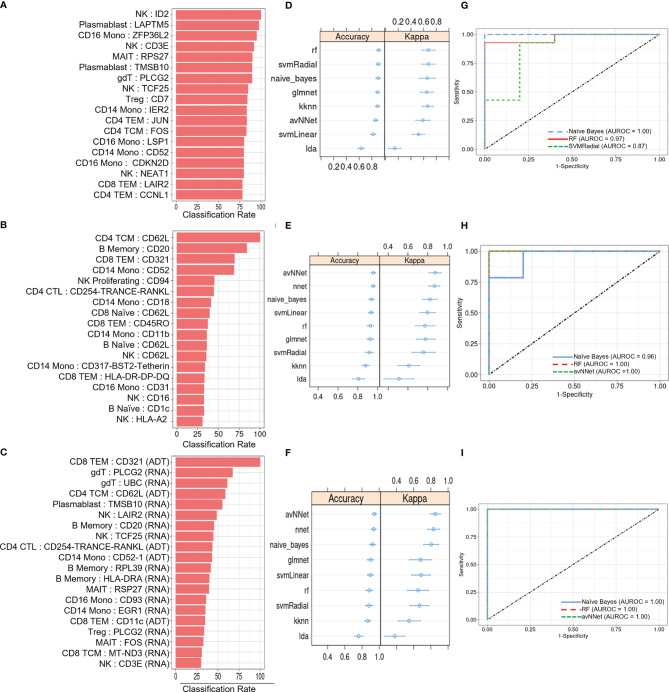
ML classification of AS and healthy subjects. Classification rate of **(A)** top 18 DEGs, **(B)** top 18 DEPs, and **(C)** top 20 features from the set of combined DEGs and DEPs from ensemble feature selection, along with their respective **(D–F)** prediction accuracy and kappa using 9 ML models and **(G–I)** ROC curves based on test set classification. Error bars indicate 95% confidence interval.

We next investigated the diagnostic potential of the cell type specific gene and protein expression differences we observed above by using these biomarkers to perform machine learning classification of AS and healthy subjects. Taking the mean normalized expression of each DE gene (**Supplementary Table 3**) or protein (**Supplementary Table 4**) across each subject’s cells in the corresponding cell types, we performed ensemble feature selection (23) using four ML algorithms to identify an optimal subset of 18 features among the DE genes and 18 features among the DE proteins with the highest classification rate. Feature importance was generally higher among DE genes than proteins (**Figures 4A, B**), and applying this approach to the combined DE gene and DE protein sets yielded a set of 20 optimal features (5 DE proteins, 15 DE genes) with classification rates similar to that of DE proteins (**Figure 4C**). Nine ML classifiers trained on each of these optimized feature sets yielded an average accuracy of 0.63 – 0.90 for DE genes, 0.76 – 0.93 for DE proteins and 0.80 – 0.96 for the set of combined features (**Figures 4D–F**), and kappa ranged between 0.61 – 0.88 for all models. For DE genes, the three best-performing models, SVMRadial, RF, and Naïve Bayes, achieved AUROCs of 0.87, 0.97, and 1.00 (**Figure 4G**), respectively, while the corresponding best three for DE proteins, Naïve Bayes, RF, and avNNet, achieved AUROCs of 0.96, 1.00, and 1.00 (**Figure 4H**). Finally, the best three models for the combined DE gene and DE protein set, RF, Naïve Bayes, and avNNet, classified all test set subjects perfectly, achieving an AUROC of 1.00 (**Figure 4I**). Similar classification accuracy was achieved by models built using scaled, within-subject counts of each DEG or DEP (**Supplementary Figure 4**), indicating that model performance is not substantially explained by our normalization approach.

## Discussion

In this study, we performed a multi-omic analysis of ankylosing spondylitis, identifying transcriptomic and surface epitope changes associated with disease. Our single-cell approach also identified cell subsets that may contribute to pathogenesis, allowing for the further elucidation of key AS pathways.

### AS PBMCs Overexpressed Genes and Proteins Linked With Inflammation

CD14^+^ monocytes in patients with AS overexpressed *CXCL8*, which encodes IL-8. The increased production of IL-8 is correlated with AS (25). *CXCL8* is also known to induce *S100A11* expression (24), which is overexpressed in the AS NK cells in our dataset. Consequently, *CXCL8* in CD14^+^ monocytes could be driving the observed increased production of IL-8 and other disease-related genes like *S100A11*. CD4^+^ T_CM_ cells in patients with AS overexpressed *KLRB1*, which is correlated with high tumor necrosis factor and interferon-γ co-expression potential in CD4^+^ memory T cells. KLRB1^+^ CD4^+^ T_CM_ cells also have increased IL-17A production (26). As a result, increased KLRB1 expression in CD4^+^ T_CM_ may upregulate inflammatory pathways and cytokines related to AS pathogenesis. CD39, an ecto-enzyme which converts extracellular ATP to extracellular adenosine, was significantly underexpressed in the Tregs of patients with AS. Tregs with low CD39 expression produce IL-17, contrary to their CD39^+^ counterparts that suppress IL-17 production (27). Consequently, low CD39 expression on AS Tregs could be associated with limited functionality and the production of inflammatory IL-17.

Both memory B cells and CD16^+^ monocytes in AS overexpressed IL-18Rα. IL-18 is a pro-inflammatory cytokine whose role in AS remains to be further elucidated with previous studies finding comparable IL-18 levels between healthy controls and AS patients (28). IL-18Rα also interacts with IL-37, which is significantly overexpressed in AS and may inhibit pro-inflammatory cytokine expression in AS PBMCs (29, 30). Consequently, increased IL-18Rα expression suggests the importance of cytokine signaling pathways in AS outside of the standard IL-23/IL-17 axis. On the other hand, both CD4^+^ T_EM_ and MAIT cells in patients with AS underexpressed IL-7Rα relative to control patients, with MAIT cells also underexpressing *IL7R*. Our result contrasts with a MAIT-specific increase in IL-7R expression previously observed in AS patients that was associated with increased IL-17 expression (31), though further studies are needed to clarify the role of IL-7 signaling in AS.

Several inflammatory pathways were observed to be significant in AS at a nominal level, including the signaling of inflammatory pathways such as IL-4/IL-13 signaling (**Supplementary Table 5**). Many of these pathways involved the upregulation of *FOS* and *JUNB*, which are also linked to the abnormal expression of *NFKB* in AS CD8^+^ T cells (32). Future studies are needed to follow up on these important inflammatory pathways.

Overall, our results suggest that a diverse set of cell types in the peripheral blood help drive the production of inflammatory cytokines. The identified surface proteins and genes could serve as potential therapeutic targets in AS.

### Differential Genes and Proteins in AS Are Linked With Other Immune-Mediated Diseases

A subset of differentially expressed genes and proteins identified in our AS dataset are important in other known immune-mediated diseases. CD8^+^ T_EM_ cells in AS overexpressed PD-1, which is a regulatory checkpoint inhibitor receptor for the immune system that has been proposed to play an important role in rheumatic disorders (33). Naïve B cells in AS overexpressed CD5. A similar result was found in a study on rheumatoid arthritis, which found that CD5^+^ B cells may be involved in autoimmunity (34). CD16^+^ monocytes in AS were seen to overexpress FR-β, which is part of a family of folate binding receptors. FR-β was upregulated in activated macrophages in the synovial tissue of patients with rheumatoid arthritis (35). Memory B cells overexpressed CCR6, which is a chemokine receptor with the ligand CCL20. CCR6 was seen to be overexpressed in the B cells of patients with systemic lupus erythematosus (SLE) (36). Naïve AS B cells also overexpressed CD74, which was similarly overexpressed in mice with an SLE phenotype (37). Naïve B cells in AS overexpressed *TCL1A*. A previous study has found that B cells in patients with Primary Sjögren’s syndrome upregulated *TCL1A* (38). These genes and surface proteins could play a similar role in AS and suggest common treatment strategies across several types of immune-mediated diseases.

CD14^+^ monocytes in patients with AS overexpressed CD52, a glycoprotein whose ligation results in T-cell activation and proliferation (39). Notably, CD52 is the therapeutic target of alemtuzumab, which is approved for the treatment of multiple sclerosis (40). Additionally, *CD52* was overexpressed across several AS cell types in our transcriptomic dataset, affirming the importance of CD52 at the transcriptome level and suggesting that *CD52* expression is upregulated in several cell types in the peripheral blood.

These results indicate that AS may share several differentially expressed genes and proteins with other immune-mediated diseases, indicating potential shared pathogenetic mechanisms and treatment strategies.

### Cell Subsets, Genes, and Proteins Examined in Past AS Studies

In our study, there was a statistically significant overrepresentation of CD16^+^CD56^dim^ NK cells in patients with AS (**Supplementary Figure 2B**). Prior studies have shown that CD16^+^CD56^dim^ NK cells exhibit increased cytotoxic activity and are overexpressed in AS (41, 42). Although previous studies report conflicting observations on whether circulating NK cell abundance is altered in AS (12), by conducting *de novo* clustering on NK cells, we identified a subcluster that was overrepresented in AS (**Supplementary Figures 2D–F**). This subset had an overexpression of CD16, along with CD161 and CD38, which have been linked to cytotoxicity and pro-inflammatory NK cell subsets respectively (43, 44). Furthermore, a gene set enrichment analysis of this cluster revealed an upregulation of natural killer cell mediated cytotoxicity (p = 0.03). As a result, this cluster could be driving inflammation in AS and could consequently be a NK subset of interest for investigating NK cell activity in AS.

CD8^+^ T_EM_ cells and NK cells in AS overexpressed genes related to cytotoxicity, including *GZMH, GZMB*, and *NKG7*. This result agrees with our finding that CD16 expression is increased in NK cells since high CD16 expression is linked with NK cell cytotoxicity (41). CD8^+^ T_EM_ cells also overexpressed both *CCL4* and *CCL4L2*, which are inflammatory chemokines. These results provide further evidence for the increased cytotoxic activity of NK cells and CD8^+^ T_EM_ cells in AS.

We compared transcription factors with p values below 0.05 against the data of a previous paper that used ATAC-seq on AS PBMCs to examine the role of transcription factors in AS (24). Our observed overexpression of GCM1, YY1, ETS1, ETV4, and ELF1 in CD4^+^ T cells was confirmed in the ATAC-seq data, where all these transcription factors were also statistically significant. GCM1 had a particularly high log fold change in the ATAC-seq dataset, suggesting that this transcription factor may be particularly important.

CD8^+^ T_EM_ cells in AS overexpressed *CMC1*, variants of which are associated with AS (45). We also observed an overexpression of *TNFSF10* in the CD16^+^ monocytes of AS patients relative to control patients. *TNFSF10* is part of the TNF superfamily and has been shown to be associated with AS pathogenesis (46). Naïve B cells in AS were observed to increase the expression of *CXCR4*. This gene has also been found to be upregulated in the hip synovial tissue of patients with AS (47).

CD14^+^ monocytes, naïve B cells, and CD16^+^ monocytes in AS displayed increased expression of *HLA-DRB5*. A previous study in the Chinese Han population found that increased DNA copy number of HLA-DQA1 but not HLA-DRB5 was associated with AS, though they did not measure transcription levels of these genes (48).

### ML Classification of AS and Healthy Subjects

We found that AS-associated differences in cell-specific gene and surface protein expression could distinguish AS from healthy subjects, based on >0.95 AUROC achieved by several machine learning algorithms (**Supplementary Table 7**), though the general performance of these models may be limited by sample size (particularly for AS subjects). We nevertheless note that transcriptomic or cell surface protein expression of CD52 was consistently identified as an important feature in DEG, DEP, or DEG and DEP feature sets used for model training, which, given its biological significance as discussed above, may warrant further investigation as a diagnostic and therapeutic target.

Besides modest cohort size, other limitations of this study include the sampling of patients from a single center and the use of only molecular biomarkers for subject classification. Future multi-center studies can address these limitations by recruiting patients with AS and with back or joint pain from similar and unrelated diseases as well as by incorporating clinical and demographic data into the classification model.

### Summary

This study has applied CITE-seq technology for the analysis of ankylosing spondylitis (AS), allowing for the important characterization of gene and cell surface proteins in AS. Numerous cell types overexpressed *CD52* on the transcriptomic and surface epitope level, which is involved in T-cell activation and is an important therapeutic target in other types of immune-mediated diseases. A pro-inflammatory NK cell subset was significantly overrepresented in AS that was characterized by high expression of CD16, CD161, and cytotoxic genes. This subset could be driving the overrepresentation of CD16^+^ CD56^dim^ NK cells, a subset of NK cells with high cytotoxic activity, that was observed in our dataset and previous studies. *CD39* was underexpressed in Tregs, whose underexpression has been linked to IL-17 production and loss of functionality. CD14^+^ monocytes in AS overexpressed *CXCL8*, which has been associated with increased inflammatory IL-8 expression.

Natural killer cells overexpressed cytotoxic genes along with *S100A11*, whose expression is induced by CXCL8. CD4^+^ T_CM_ cells in AS have a high expression of *KLRB1*, which is related to TNF and IFN-*γ* co-expression potential as well as IL-17A production. Memory B cells and CD16^+^ monocytes overexpressed IL-18R*α*, which interacts with the cytokines IL-18 and IL-37. CD5 was overexpressed in AS naïve B cells with CD5^+^ B cells being known to be involved in autoimmunity.

Together, these results suggest cell type-specific changes both on the RNA level and on the surface protein level that may elucidate the pathogenesis of AS. The high classification rate of machine learning classifiers based on these gene and protein differences further indicates their potential as diagnostic biomarkers.

## Data Availability Statement

The datasets presented in this study can be found in online repositories. The names of the repository/repositories and accession number(s) can be found at https://www.ncbi.nlm.nih.gov/geo/, GSE194315.

## Ethics Statement

The studies involving human participants were reviewed and approved by University of California, San Francisco IRB. The patients/participants provided their written informed consent to participate in this study.

## Author Contributions

WL and LG conceived and supervised the project. DP, JH, H-WC, TB, LG, and WL recruited study subjects and performed clinical annotation. Z-MH performed experimental procedures. SA, SK, JL, and WL performed data analysis. SA, SK, JL, LG, and WL wrote and revised the manuscript. All authors contributed to the article and approved the submitted version.

## Funding

This study was funded by grants from the National Psoriasis Foundation to WL. This study was also supported by PREMIER, a NIH/NIAMS P30 Center for the Advancement of Precision Medicine in Rheumatology at UCSF (P30AR070155​). WL is supported by NIH grant R01AR078688. JL is supported by NIH grant T32AR007175.

## Conflict of Interest

WL has received research grant funding from Abbvie, Amgen, Janssen, Leo, Novartis, Pfizer, Regeneron, and TRex Bio. LG has received research/grant support from Novartis, Pfizer and UCB and consulting fees from AbbVie, Eli Lilly, Gilead, Janssen, Novartis, Pfizer and UCB.

The remaining authors declare that the research was conducted in the absence of any commercial or financial relationships that could be construed as a potential conflict of interest.

## Publisher’s Note

All claims expressed in this article are solely those of the authors and do not necessarily represent those of their affiliated organizations, or those of the publisher, the editors and the reviewers. Any product that may be evaluated in this article, or claim that may be made by its manufacturer, is not guaranteed or endorsed by the publisher.

## Supplementary Material

Supplementary Table 1 Clinical and demographic characteristics of study subjects.

Supplementary Table 2 TotalSeq Antibodies.

Supplementary Table 3 Differentially expressed genes between AS and healthy controls.

Supplementary Table 4 Differentially expressed proteins between AS and healthy controls.

Supplementary Table 5 Gene and protein markers of *de novo* NK cell clusters.

Supplementary Table 6 Gene set enrichment analysis between AS and healthy controls.

Supplementary Table 7 ML classification performance.
