# Supplementary material for: Single Cell Transcriptome and Surface Epitope Analysis of Ankylosing Spondylitis Facilitates Disease Classification by Machine Learning
Source: Front Immunol. 2022 May 13;13:838636. doi: 10.3389/fimmu.2022.838636 (PMC9135966; doi:10.3389/fimmu.2022.838636)
Supplement: Supplementary file 8 [file DataSheet_1.pdf]

Supplementary Figure 1

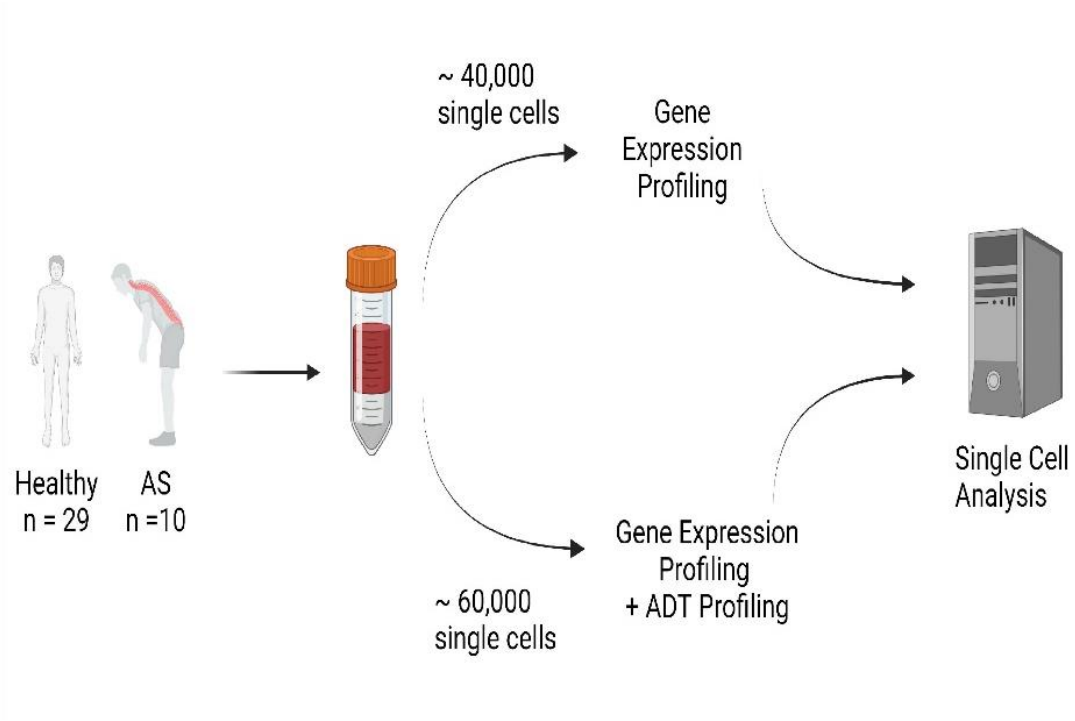

Supplementary Figure 1. Study design.

Supplementary Figure 2

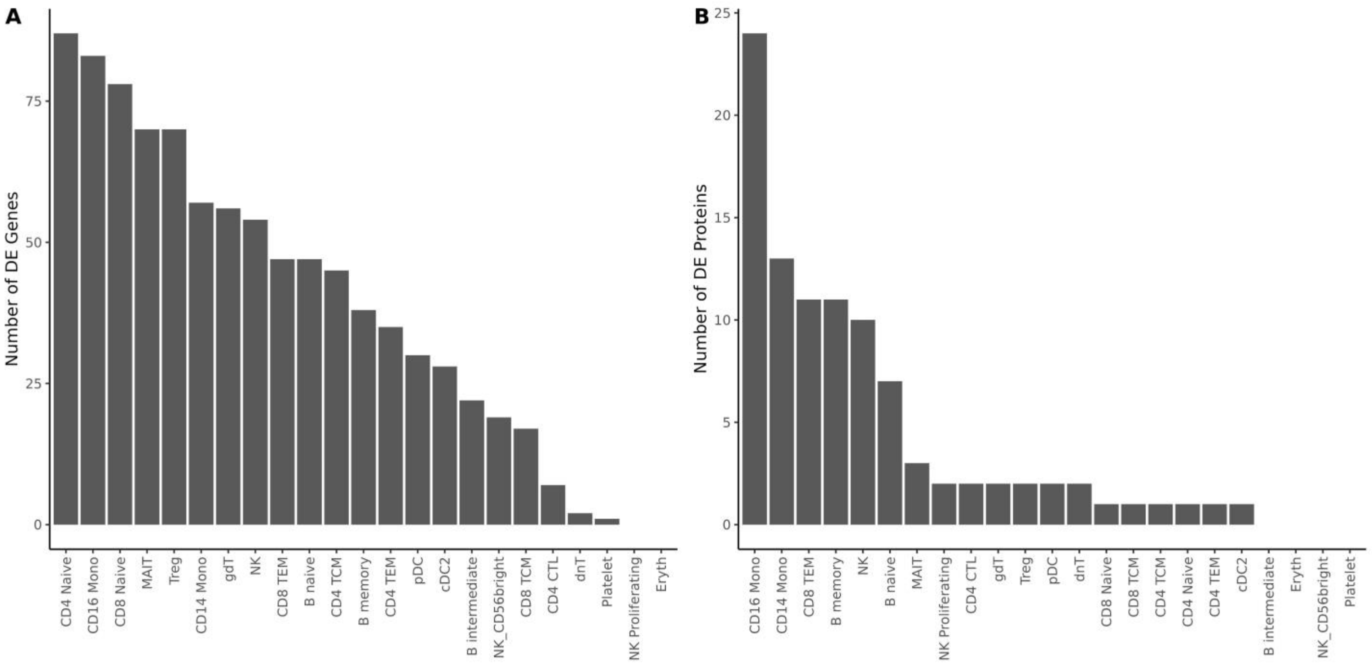

Supplementary Figure 2. Features with cell-type specific expression differences associated with AS. (A) Number of DEGs per cell type. **(B)** Number of DEPs per cell type.

Supplementary Figure 3

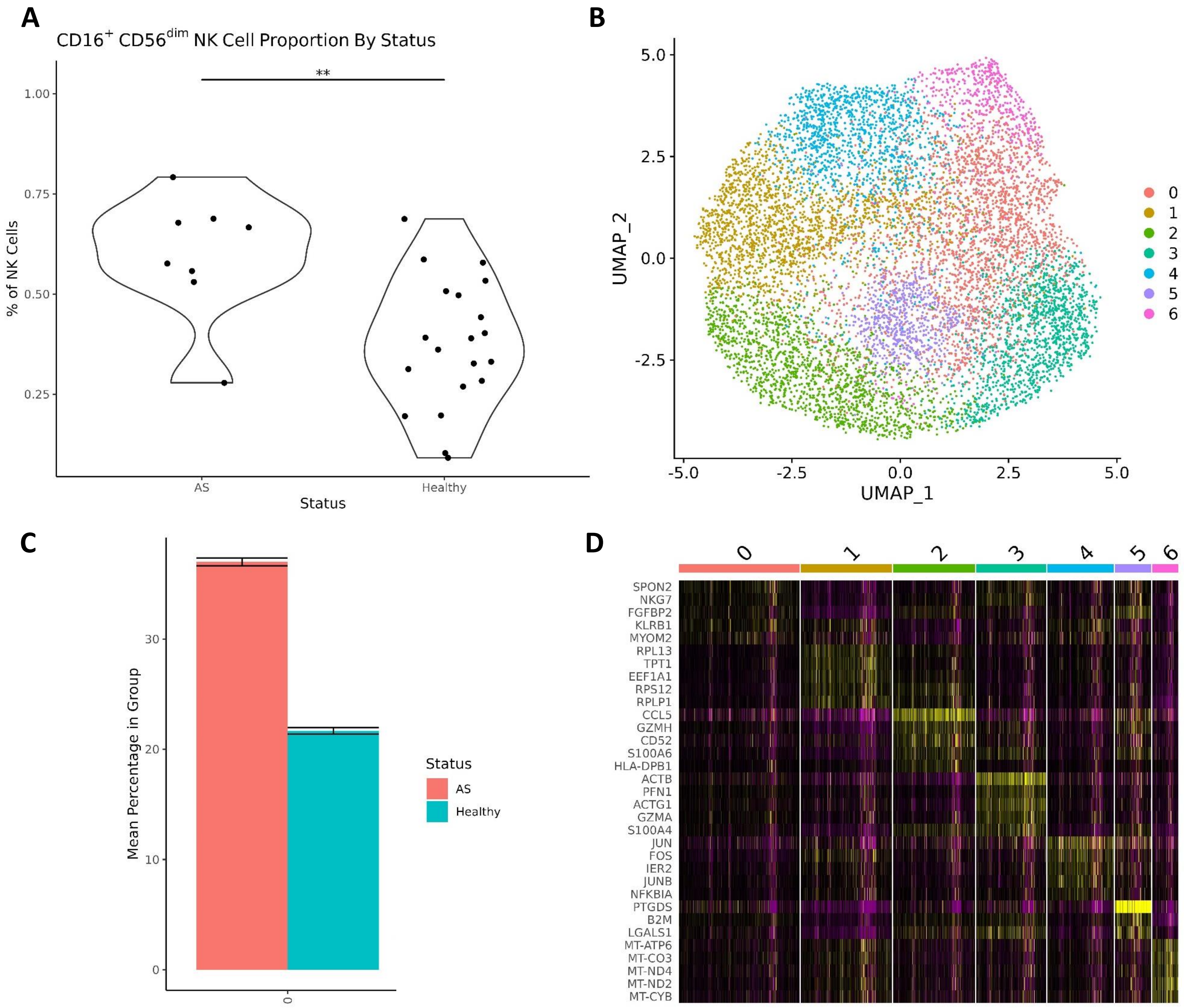

Supplementary Figure 3. NK cell subset analysis. identified by de novo clustering. (A) The mean percentage of CD16$^+$CD56^{dim} cells out of total NK cells in each subject, averaged across AS and across healthy groups. 2 patients with fewer than 10 CD16$^+$CD56^{dim} NK cells are not shown. ** indicates Wilcoxon rank sum test p-value = 0.006. **(B)** UMAP of NK clusters. **(C)** Mean percentage of Cluster 0 NK cells (out of total NK cells in each subject) between AS and healthy subjects. **(D)** Heatmap of top 5 RNA markers in each NK cluster.

Supplementary Figure 4

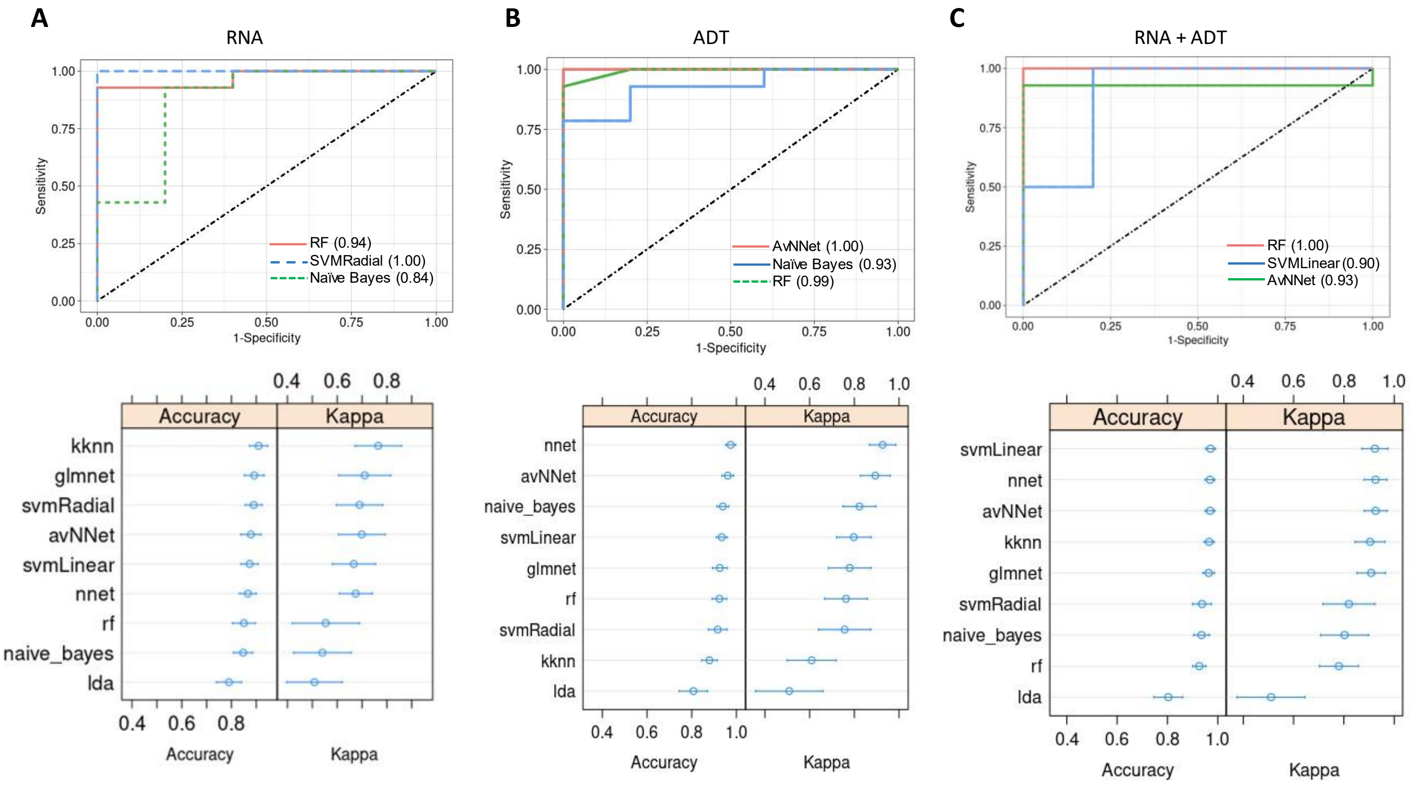

Supplementary Figure 4. Machine learning classification of AS and healthy subjects based on within-subject normalized counts. Feature expression normalized across cells within each subject were input to machine learning classification based on the top 20 features among **(A)** DEGs, **(B)** DEPs, and **(C)** both DEGs and DEPs. ROC curves based on test set classification. Error bars indicate 95% confidence interval.
